# Development of the R263K Mutation to Dolutegravir in an HIV-1 Subtype D Virus Harboring 3 Class-Drug Resistance

**DOI:** 10.1093/ofid/ofy329

**Published:** 2018-12-12

**Authors:** N Ahmed, S Flavell, B Ferns, D Frampton, S G Edwards, R F Miller, P Grant, E Nastouli, R K Gupta

**Affiliations:** 1 Mortimer Market Centre, Central and North West London NHS Foundation Trust, London, United Kingdom; 2 Division of Infection and Immunity, University College London, London, United Kingdom; 3 Clinical Research Department, Faculty of Infectious and Tropical Diseases, London School of Hygiene and Tropical Medicine, London, United Kingdom; 4 Centre for Clinical Research in Infection and Sexual Health, Institute for Global Health, University College London, London, United Kingdom; 5 Department of Clinical Virology, University College London Hospitals NHS Foundation Trust, London, United Kingdom; 6 Department of Population, Policy and Practice, UCL GOS Institute of Child Health, London, United Kingdom

**Keywords:** adolescents, ARVs, dolutegravir, HIV, resistance

## Abstract

Dolutegravir (DTG), a second-generation integrase strand-transfer inhibitor (INSTI), is equivalent or superior to current non-nucleotide reverse transcriptase inhibitors (NNRTIs), protease inhibitors (PIs), and first-generation INSTI-based antiretroviral regimens (ARVs). It has the potential to make big improvements in HIV control globally and within patients. This is perhaps the most “precious” HIV drug available. The integrase mutation R263K has been observed in tissue culture experiments and in patients treated with dolutegravir monotherapy in clinical trials. Globally, adherence and monitoring may be less than optimal and therefore DTG resistance more common. This is particularly important in low–middle-income countries, where patients may remain on failing regimens for longer periods of time and accumulate drug resistance. Data on this mutation in non–subtype B infections do not exist. We describe the first report of the R263K integrase mutation in a dolutegravir-exposed subtype D–infected individual with vertically acquired HIV. We have used deep sequencing of longitudinal samples to highlight the change in resistance over time while on a failing regimen. The case highlights that poorly adherent patients should not be offered dolutegravir even as part of a combination regimen and that protease inhibitors should be used preferentially.

Dolutegravir (DTG), a second-generation integrase strand-transfer inhibitor (INSTI), is equivalent or superior to current non-nucleotide reverse transcriptase inhibitors (NNRTIs), protease inhibitors (PIs), and first-generation INSTI-based antiretroviral regimens (ARVs) [1]. DTG has been shown to have excellent efficacy, tolerability, few drug–drug interactions, and has the potential to reduce pill burden.

DTG appears to have a high genetic barrier to resistance, unlike the other drugs within the INSTI class, raltegravir and elvitegravir, which select for major resistance mutations such as N155H, Y143H/R/C, G140A/S, and Q148H/R/K. DTG retains activity in the face of these mutations, although mutations at G140 and Q148 together can result in significant drug resistance to DTG [2, 3].

Treatment-naïve patients taking combination ARV regimens containing dolutegravir who experience virological failure are rarely found to have mutations in the *integrase* gene [4]. Dolutegravir monotherapy in naïve patients, on the other hand, is associated with more frequent selection of drug resistance mutations such as R263K, G118R, S230 [2], and possibly resistance mutations outside the integrase gene [5, 6]. In treatment-experienced patients, DTG resistance is also observed, most commonly in those previously treated with raltegravir [7, 8], although not exclusively [4]. A number of additional mutations observed in patients can increase DTG resistance, including L74M and E138K [9, 10].

The integrase mutation R263K confers moderate resistance to DTG with a significant reduction of in vitro replication fitness [11]. It has been observed in treatment-naïve patients by ultradeep sequencing, in experienced patients [4], and recently as transmitted drug resistance [12]. Most reports of the R263K mutation stem from subtype B–infected individuals in high-income settings treated with ABC/3TC/DTG or DTG monotherapy. In low–middle-income settings, R263K and other DTG resistance mutations may be more common where patients remain on failing regimens for longer periods of time and use alternate NRTIs temporarily due to stockouts or undisclosed ARV use, thereby accumulating multi-NRTI resistance [13–15].

We describe the first report of the R263K integrase mutation in a dolutegravir-exposed subtype D–infected individual with vertically acquired HIV.

## CASE REPORT

A 22-year-old East African woman with vertically acquired HIV had been diagnosed shortly after birth. Her baseline viral load (VL) was 375 000 copies/mL, her CD4 was 150 cells/mm^3^, and she had subtype D infection. At diagnosis, zidovudine monotherapy was commenced. Didanosine was added 2 years later, and she was switched to stavudine, lamivudine, and nelfinavir at 3 years of age. The VL dropped to 700 copies/mL; however, it rebounded to 6000 copies/mL: at that time, a first resistance test showed M184V and D30N mutations. The patient then received zalcitabine, abacavir, and amprenavir. Subsequently, she maintained poor virological control despite changing antiretrovirals three times, with NNRTIs introduced during these changes (Table 1). Poor adherence continued until 11 years of age, when virological suppression was achieved with maraviroc, etravirine, and twice-daily darunavir/ritonavir. Subsequently, she disengaged from care, with inconsistent attendance over a period of 8 years. On re-engagement in care, her VL was 1610 copies/mL, and her CD4 was 104 cells/mm^3^. At that time, resistance testing showed NRTI (M184V, T69D, T215S, D67N, K219Q), NNRTI (Y181C, Y188L, H221Y) and PI (L10I, D30N, K20T, L33F, K43T, N88D) resistance, with PI resistance to nelfinavir. Integrase polymorphisms (17N, 256E, 112V, 113V, 201I, 234I) were detected. Maraviroc, etravirine, and darunavir/ritonavir (twice daily) were restarted. This regimen was simplified to darunavir/ritonavir and maraviroc, and subsequently to darunavir/ritonavir monotherapy once virological suppression was achieved. Six months later, the VL rebounded to 8600 copies/mL, and DTG 50 mg once a day was added. Poor engagement continued for 18 months; at this later, time integrase resistance testing showed the R263K mutation conferring low-level resistance to DTG and raltegravir, with intermediate resistance to elvitegravir. R263K was confirmed by next-generation sequencing (NGS) using an analysis percentage minority variant threshold of >20%. To avoid accumulation of integrase resistance mutations with ongoing poor adherence, she was switched to tenofovir, darunavir/ritonavir. Follow-up NGS sequencing 3 months after the first resistance test showed the R263K mutation at <5% in a sample with a VL of 61 000 copies/mL.

**Table 1.** Summary of Antiretroviral History

Age, y	Antirsetrovirals	VL on Starting ARVs	VL After Starting ARVs	Resistance Test on Regimen
0	AZT	375 000	-	
2	AZT, DDI	-	375 000	
3	D4T, 3TC, NFV	-	700	M184V, D30N
4	DDC, ABC, AMP	6000	-	
6	D4T, DDI, NVP	-	31 000	
8	DDI, EFV, NVP	17 000	25 000	
10	TIP, TDF, FTC	34 000	<50	
18	MVC, ETV, DRV/RIT	1610		M184V, T69D, T215S, D67N, K219Q, Y181C, Y188L, H221Y, L10I, D30N, K20T, L33F, K43T, N88D
	MVC, DRV/RIT	-	<50	
	DRV/RIT	<50		
19	DRV/RIT, DTG (OD)	8600		R263K INT 50.8%, L33F PR 99.7%, N88D PR 99.7%, D30N PR 99.9%, K43T PR 98.8%, D67N RT 92.3%, T215S RT 99.6%, K219Q RT 99.7%, T69D RT 99.8%, Y181C RT 99.8%, Y188L RT 99.8%, H221Y RT 99.7%
20	DRV/RIT, TDF	99 000		R263K INT 20.7%, K20T PR 99.7%, L33F PR 99.7%, N88D PR 99.8%, D30N PR 99.8%, K43T PR 99.7%, D67N RT 90.1%, T215S RT 99.6%, K219Q RT 99.8%, T69D RT 98.2%, Y181C RT 99.8%, Y188L RT 99.8%, H221Y RT 99.8%

% refers to abundance by ultradeep sequencing for the last 2 time points.

Abbreviations: 3TC, lamivudine; ABC, abacavir; AMP, amprenavir; AZT, zidovudine; D4T, stavudine; DDC, zalcitabine; DDI, didanosine; DRV/RIT, darunavir/ritonavir; EFV, efavirinez; ETV, etravirine; FTC, emtricitabine; INT, intergrase; MVC, maraviroc; NFV, nelfinavir; NGS, next-generation sequencing; NVP, nevirapine; OD, once a day; PI, protease inhibitor; RT, reverse transcriptase; TDF, tenofovir; TIP, tipranavir.

Reasons for poor adherence and disengagement over time included drug adverse reactions and pill burden, a lack of family support, and lack of finances to attend outpatient appointments. The patient reported low mood, which reduced her motivation to take ARVs and engage in care. Despite multiple strategies to facilitate adherence, this patient declined psychological and mental health support.

## DISCUSSION

The World Health Organization has recommended that countries consider a change from efavirenz-based regimens to dolutegravir-based regimens where pretreatment drug resistance to NNRTI has exceeded 15% [16, 17]. If DTG scale-up is to occur, drug resistance to DTG in different HIV subtypes needs to be monitored. Although at present significant DTG resistance in sub-Saharan populations is very rare [18], it has been documented recently in a heavily experienced patient who had previously failed raltegravir. We report occurrence of the R263K integrase mutation 18 months into treatment with DTG in the context of vertically acquired subtype D infection. This mutation is known to reduce viral fitness, and its loss was associated with an increase in viral load [19]. Further surveillance for dolutegravir resistance is warranted globally.
